# The Lipid Enigma: A Case Report Highlighting Diagnostic Challenges in Adipocytic Tumors

**DOI:** 10.7759/cureus.71233

**Published:** 2024-10-10

**Authors:** Vikas Dhupar, Jochima E Cota, Anita E Spadigam, Anita Dhupar, Nairica Rebello

**Affiliations:** 1 Oral and Maxillofacial Surgery, Goa Dental College and Hospital, Panjim, IND; 2 Oral and Maxillofacial Pathology, Microbiology, and Forensic Odontology, Goa Dental College and Hospital, Panjim, IND

**Keywords:** atypical lipomatous tumor, immunohistochemistry, infiltrating lipoma, mandible, mdm2 amplification

## Abstract

Infiltrating lipomas are a rare form of lipomas exhibiting unusual clinical behavior. We report a case of an adipocytic tumor of a 31-year-old male diagnosed with an infiltrating lipoma in the right submandibular region. It exhibits unusual clinical features such as invasion into surrounding structures, posing significant diagnostic challenges. The histopathological findings and differential diagnosis for this case are discussed, underscoring the importance of investigation techniques for accurate diagnosis and treatment planning. Infiltrating lipomas, while benign, can mimic malignant adipocytic tumors such as atypical lipomatous tumors (ALTs) and well-differentiated liposarcomas (WDLS), complicating diagnosis. This case underscores the importance of combining histopathology, immunohistochemistry (IHC), and molecular testing, particularly murine double minute clone 2 (MDM2) amplification, to distinguish between benign and malignant tumors.

## Introduction

Adipocytic tumors encompass a diverse spectrum of neoplasms, ranging from benign lipomas to aggressive liposarcomas [1]. While the majority are benign, certain variants can exhibit atypical features that complicate diagnosis and treatment. These tumors have a propensity to invade surrounding tissues and demonstrate a tendency for recurrence, necessitating careful management [2]. This report presents a case that illustrates the diagnostic complexities associated with uncommon adipocytic tumors.

## Case presentation

**Figure 1:**
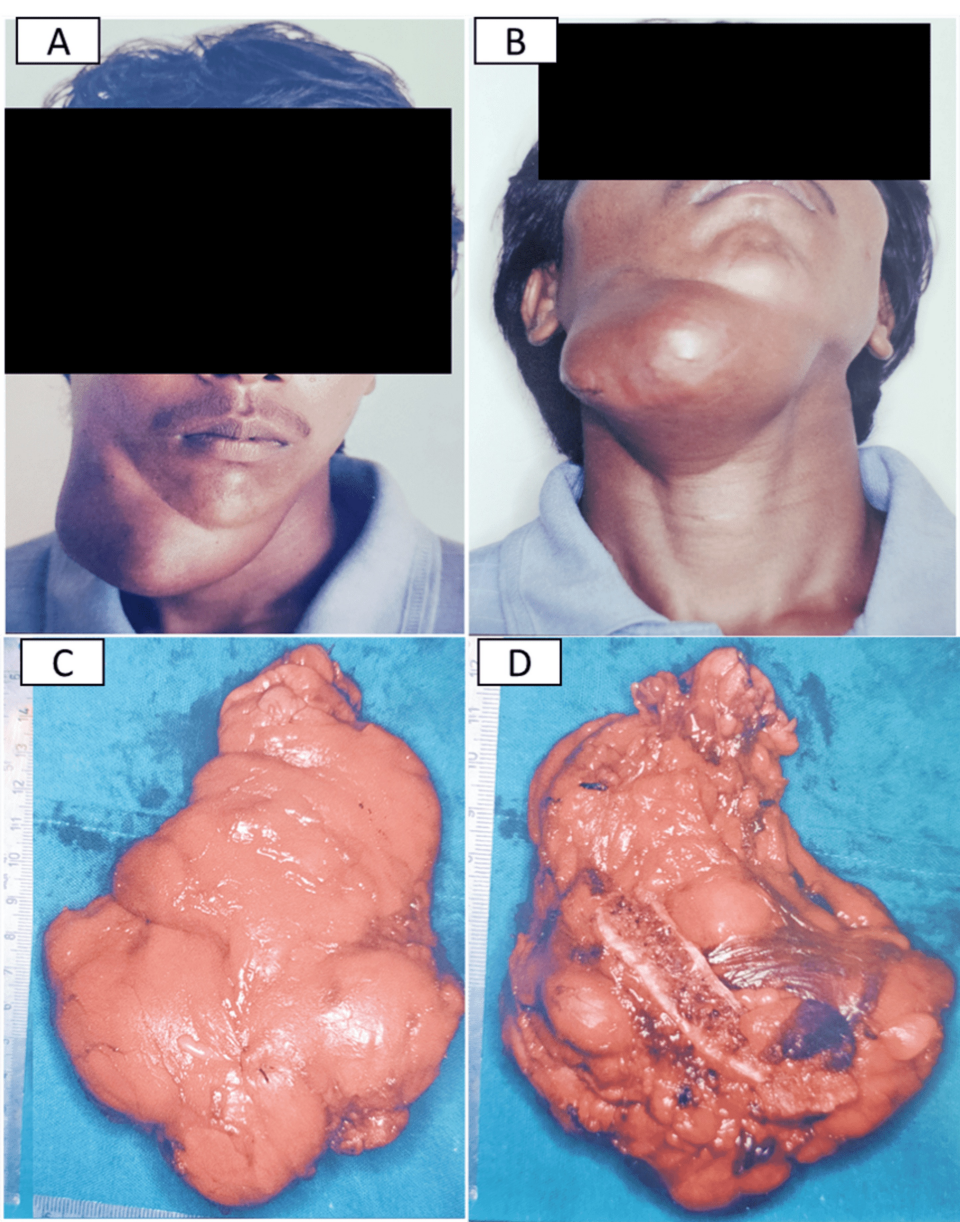
(A and B) Clinical images showing submandibular swelling. (C and D) Gross of the excised specimen

**Figure 2:**
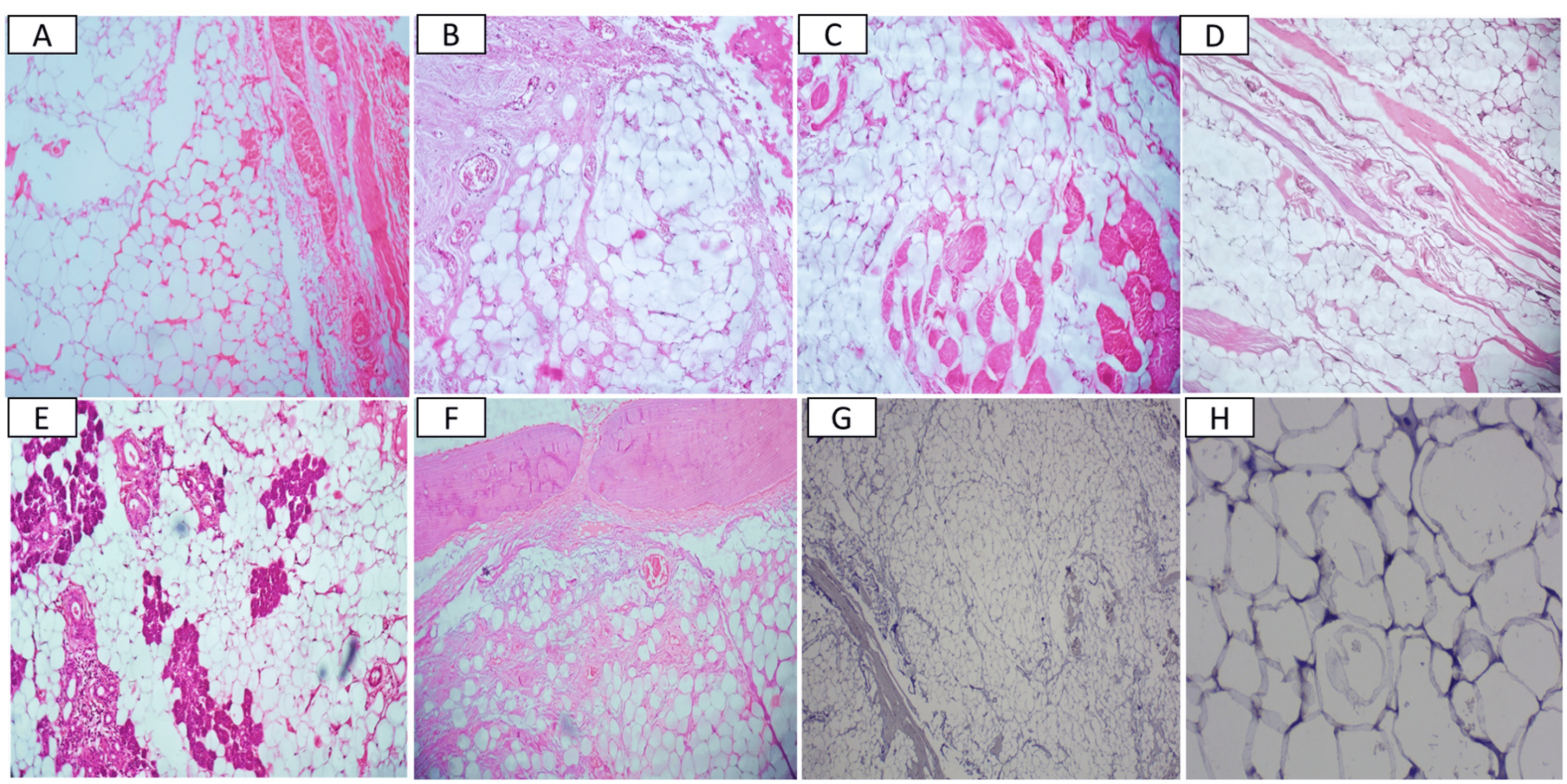
(A and B) Diffuse sheets of adipocytes with no evidence of atypia (hematoxylin and eosin {H&E}: 10×). (C and D) Intra- and intermuscular infiltration (H&E: 10×). (E) Infiltration into the salivary gland (H&E: 10×). (F) Cortical bone erosion (H&E: 10×). (G and H) Immunohistochemistry for MDM2 showing negative expression (H&E: 10× and 40×) MDM2: murine double minute clone 2

A 31-year-old male, laborer by profession, presented with an eight-year history of swelling in the right submandibular region (Figure 1). The patient was apparently alright eight years prior when he first noticed a pea-sized swelling on the right side of the face but did not get it treated as it was asymptomatic. The swelling gradually increased in size to reach the present size of 14×10 cm. However, in the last six months, the patient complained of pain in relation to the right lower mandible and hence decided to visit the hospital. On examination, the mass was non-tender, non-fluctuant, and fixed to the mandible. A computed tomography (CT) scan revealed ill-defined expansile masses and a hazy, amorphous, hypodense lesion in the right submandibular region, with an erosion of the cortical plate of the mandible. A differential diagnosis of a benign salivary gland neoplasm and a benign mesenchymal neoplasm of fibroblast, muscle, nerve, and adipocytes was considered. An incisional biopsy from the submandibular region revealed sheets of normal-appearing adipocytes; hence, a wide local excision and mandibular osteotomy were planned. The histopathological analysis (Figure 2) of the excisional tissues showed diffuse sheets of normal-appearing adipocytes with no atypia. There was evidence of inter- and intramuscular and salivary gland infiltration along with an erosion of the cortical bone of the mandible. The absence of murine double minute clone 2 (MDM2) amplification was confirmed by the negative immunohistochemistry (IHC) staining (Figure 2G, 2H), thus confirming the benign nature of the tumor, leading to a diagnosis of an infiltrating lipoma. The patient experienced no postoperative complications, with healing progressing satisfactorily. During the five-year follow-up period, no signs of recurrence were reported.

## Discussion

Infiltrating lipomas constitute less than 1% of all lipomas and exhibit unusual clinical behavior, including recurrence and aggressive local invasion, which necessitated a thorough exploration of its pathogenesis. Intramuscular lipomas can develop across all age groups, from childhood to old age, but they are most commonly seen in individuals aged 40-70 years. There is no consensus on a clear gender predilection. In the head and neck region, various muscles have been reported to be affected, including the neck muscles, tongue, cheek, orbicularis, and temporalis muscles. Among these, the tongue is one of the most frequently involved sites [3].

The development of adipocytic tumors has been subject to various theories. Historically, the hypertrophy theory proposed that these tumors arise from the simple enlargement of preexisting fat cells. However, this theory has been largely discredited and is not accepted due to its inability to explain the neoplastic characteristics of these lesions. A more widely accepted explanation is the metaplasia theory, which suggests that adipocytic tumors result from the aberrant differentiation of in situ mesenchymal cells. According to this theory, primitive mesenchymal cells within the tissue may undergo metaplasia, differentiating into adipocytes under certain conditions [4]. An alternative theory states that dormant congenital lesions could be triggered into growth by external factors such as hormonal changes, trauma, or inflammation [5].

Ramos-Pascua et al. suggested that body mass index may be related to the development of intramuscular lipomas as two-thirds of their cases were in overweight or obese individuals [6]. Mori et al. suggested that type-selective muscular degeneration and endomysial fatty growth from atrophy may influence the infiltrative behavior of intramuscular lipomas. As muscles atrophy, the resulting fatty infiltration could provide a supportive environment for lipoma expansion, potentially enhancing its infiltrative characteristics and making it appear more aggressive [7].

To distinguish infiltrating lipomas from other adipocytic tumors, a thorough differential diagnosis is critical. One of the main challenges in diagnosing infiltrating lipomas lies in distinguishing them from atypical lipomatous tumors (ALTs), well-differentiated liposarcomas (WDLS), and other malignancies such as liposarcomas [2]. Infiltrating lipomas, though benign, can mimic the aggressive and infiltrative patterns seen in malignant adipocytic tumors due to their ability to invade adjacent muscle, bone, and glandular tissues, as seen in this case. Atypical lipomatous tumors and well-differentiated liposarcomas share morphological similarities with infiltrating lipomas, such as the presence of mature adipocytes, but often display cellular atypia and variations in nuclear size [8]. MDM2 amplification, a key diagnostic marker, plays a pivotal role in differentiating benign lipomatous tumors from malignant counterparts. MDM2, an oncogene located on chromosome 12q13-15, is frequently amplified in well-differentiated liposarcomas and atypical lipomatous tumors [9]. Its absence in infiltrating lipomas, as demonstrated in the current case, supports the benign nature of the lesion. Recurrence rates following treatment have been reported in the literature to range from 3% to 62.5%. Recurrence is often attributed to the incomplete excision of the lipoma, which may occur due to the tumor's close proximity to critical anatomical structures or concerns about potential functional impairments that could arise from fully resecting the affected muscle [3].

## Conclusions

Infiltrating lipomas, while benign, can mimic malignant adipocytic tumors such as ALTs and WDLS, complicating diagnosis. This case underscores the importance of combining histopathology, immunohistochemistry, and molecular testing, particularly MDM2 amplification, to distinguish between benign and malignant tumors. Accurate diagnosis is crucial for appropriate treatment, minimizing recurrence, and avoiding unnecessary aggressive interventions.
